# Extensive‐stage small cell lung cancer: Is prophylactic cranial irradiation necessary in the era of immunotherapy with MRI surveillance?

**DOI:** 10.1002/pro6.1200

**Published:** 2023-06-21

**Authors:** Yuanhu Yao, Nan Yao, Zhaohui Qin, Ji Ma, Jiaying Lu, Li Cui, Wanxi Qu, Shiwang Yuan, Shaodong Tong, Na Li, Hao Li

**Affiliations:** ^1^ Department of Radiation Oncology The Affiliated Wuxi People's Hospital of Nanjing Medical University Wuxi Jiangsu China; ^2^ Graduate School of Xuzhou Medical University Xuzhou Jiangsu China; ^3^ Research Center for Medical and Health Emergency Rescue Xuzhou Medical University Xuzhou Jiangsu China; ^4^ Department of Radiation Oncology The Third People's Hospital of Xuzhou Xuzhou Jiangsu China; ^5^ Department of Radiation Oncology Xuzhou Central Hospital Xuzhou Jiangsu China; ^6^ Department of Oncology Xuzhou Central Hospital Xuzhou Jiangsu China

**Keywords:** brain metastases, extensive‐stage small cell lung cancer, immunotherapy, magnetic resonance imaging, prophylactic cranial irradiation

## Abstract

**Objective:**

The role of prophylactic cranial irradiation (PCI) in treating extensive‐stage small‐cell lung cancer (ES‐SCLC) has been controversial. This study aimed to comprehensively analyze the efficacy of PCI for the treatment of ES‐SCLC under active brain magnetic resonance imaging (MRI) surveillance.

**Methods:**

Patients with ES‐SCLC with no brain metastases (BM) confirmed by MRI at the time of diagnosis who responded well to first‐line chemoimmunotherapy at three general hospitals were retrospectively included. Overall survival (OS), progression‐free survival (PFS), and cumulative incidence of BM were compared between patients who underwent PCI and those who did not.

**Results:**

In total, 66 consecutive patients treated between March 2019 and December 2021 were included in our dataset. Seventeen patients underwent PCI (PCI group) and 49 patients did not (non‐PCI group). In comparison with the non‐PCI group, PCI did not provide OS (median OS: 18.53 *vs*. 17.35 months, *p* = 0.28) or PFS (median PFS: 8.61 *vs*. 7.56 months, *p* = 0.41) benefits. When death was counted as a competing risk, the difference in the cumulative incidence rate of BM was not statistically significant (1‐year: 12.79% *vs*. 38.09%; *p* = 0.14).

**Conclusion:**

Compared to active MRI surveillance, first‐line chemoimmunotherapy followed by PCI did not improve the prognosis of patients with ES‐SCLC. Further studies are warranted to evaluate the therapeutic effects of PCI following chemoimmunotherapy.

## Graphical abstract

In the modern era of immunotherapy prolonging overall survival and increasing routine use of surveillance MRI, the use of prophylactic cranial irradiation after first‐line chemotherapy and immunotherapy was not associated with an overall survival benefit or a progression‐free survival benefit for patients with extensive‐stage small‐cell lung cancer. The use of prophylactic cranial irradiation was also not associated with a decrease in the risk of developing new brain metastases.

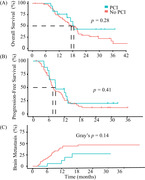

## 1 INTRODUCTION

Small‐cell lung cancer (SCLC) is an aggressive neuroendocrine cancer that grows rapidly and has a high risk of early metastasis. Approximately 15–33% of patients with SCLC have brain metastases (BM) upon initial diagnosis, and 80% of patients develop BM within 2 years.^1^, ^2^, ^3^, ^4^ Prophylactic cranial irradiation (PCI) is the standard method used for treating patients with limited‐stage SCLC that have shown a complete response to chemoradiotherapy.^5^, ^6^ However, the value of PCI in patients with extensive‐stage SCLC (ES‐SCLC) is in debate.^7^, ^8^ The randomized trial initiated by the European Organization for Research and Treatment of Cancer Radiation Oncology Group and Lung Cancer Group reported that PCI prolonged ES‐SCLC patient survival and reduced the incidence of BM.^7^ With opposing conclusions, the Japanese randomized trial demonstrated no reduction in overall survival (OS) when PCI was replaced by active magnetic resonance imaging (MRI) surveillance.^8^

More recently, two randomized phase III trials (IMpower133 and CASPIAN) indicated that anti‐programmed cell death ligand 1(PD‐L1) therapy (atezolizumab or durvalumab) significantly improved OS with a tolerable safety profile in patients with ES‐SCLC.^9^, ^10^, ^11^ Based on the outstanding findings, anti‐PD‐L1 immunotherapy plus chemotherapy has become the new first‐line standard‐of‐care for the treatment of ES‐SCLC. However, PCI was not mandated in the IMpower 133 trial, which was administered to only over 11% of patients enrolled, and no rates of BM or neurological death were reported. PCI is also poorly controlled in CASPAIN, is only available to patients undergoing chemotherapy alone, and is only delivered to 8% of the patients. There are limitations to our understanding of the benefits of introducing immunotherapy to PCI because of the lack of proper controls in the aforementioned randomized trials.

In this era of immunotherapy, no prospective studies have demonstrated the benefits of PCI in patients with ES‐SCLC. In view of these concerns, we performed this multi‐institutional real‐world study to investigate the efficacy of PCI for patients with ES‐SCLC who responded well to initial chemoimmunotherapy in the presence of active brain MRI surveillance.

## 2 MATERIALS AND METHODS

### 2.1 Study design and patient population

Information on patient demographics, cancer characteristics, treatment, and survival outcomes at three general hospitals (The Affiliated Wuxi People's Hospital of Nanjing Medical University, The Third People's Hospital of Xuzhou, Xuzhou Central Hospital) were retrospectively extracted from medical records between March 2019 and December 2021. Patients were eligible for study inclusion if 1) they were aged 18 years or older with histologically or cytologically confirmed ES‐SCLC according to the Veterans Administration Lung Study Group staging system;^12^ 2) brain MRI was performed at diagnosis and after completing first‐line chemoimmunotherapy to rule out BM; 3) they had an Eastern Cooperative Oncology Group performance status (ECOG PS) of 0–2; and 4) they had a complete or partial response to initial chemoimmunotherapy. The main exclusion criteria were as follows: 1) combination with other malignancies; 2) chemotherapy cycles <4; 3) first‐line immunotherapy using immunological agents other than atezolizumab or durvalumab; 4) disease progression during the initial chemoimmunotherapy; and 5) inadequate follow‐up data.

### 2.2 Treatment and follow up

All patients received at least four cycles of platinum plus etoposide every 3–4 weeks with either atezolizumab (administered intravenously at a dose of 1,200 mg on day 1 of each cycle) or durvalumab (administered intravenously at a dose of 1,500 mg on day 1 of each cycle). Maintenance therapy with 1,200 mg atezolizumab every 3 weeks or 1,500 mg durvalumab every 4 weeks was continued until disease progression, unacceptable toxicity, or at the physician's discretion.

During the maintenance phase, patients in the PCI group received cranial radiation at a total dose of 25 Gy delivered in 10 daily fractions (2.5 Gy per fraction) using intensity‐modulated radiotherapy with hippocampal avoidance. PCI was initiated within 8 weeks of chemotherapy. MRI monitoring of the intracranial status was conducted at least once every 2–3 months in all patients, regardless of whether PCI was performed. Consolidative thoracic radiotherapy for patients with a good response to the initial chemoimmunotherapy and palliative radiotherapy for sites other than the thorax for symptom alleviation were permitted for both groups. Treatment for intracranial relapse and other post‐progression treatments in the two groups were left to the treating physician's discretion.

### 2.3 Outcomes and evaluation method

OS was the primary endpoint and defined as the time from pathological diagnosis to death from any cause. Secondary endpoints included PFS (time from pathological diagnosis to disease progression or death) and occurrence of BM. Using the pathological diagnosis date as the index date, all enrolled patients were followed up until death or censored at the date of the last follow‐up (December 19, 2022).

Patients were required to undergo a baseline evaluation before treatment, including physical examination, laboratory tests, thoracoabdominal CT, and brain MRI scans. Treatment response was assessed using the immune response evaluation criteria in solid tumors (iRECIST) every two cycles during first‐line chemotherapy and within 8 weeks after the completion of chemotherapy. After completing the full course of treatment, patients underwent radiographic assessment every 2 months for a period of 2 years, and every 6 months thereafter. Additional CT, MRI, bone scan, positron emission tomography, or pulmonary function tests may be performed between scheduled scans if clinically indicated.

### 2.4 Statistical analysis

The chi‐square test and Fisher's exact test were used to compare categorical variables, and the Student's *t*‐test was used to test continuous variables. OS and PFS were estimated using the Kaplan‐Meier method, and group distributions were compared using log‐rank tests. The Cox proportional hazards regression model was used for the univariate and multivariate survival analyses. If the univariate analysis indicated a possible association with the outcome (*p* < 0.15), the variables were included in the multivariate analysis. A Grambsch‐Therneau test and Schoenfeld residuals were used to verify the proportional hazards assumption in the Cox analysis. We calculated the cumulative risk of BM over time using cumulative incidence curves and compared the two groups using the Gray's test. Death without evidence of brain metastasis was considered a competing risk factor. All reported *p* values for clinical endpoints were two‐sided; a *p* value of less than 0.05 is considered statistically significant. All statistical analyses were performed using the SPSS (version 22.0; IBM) and R (version 4.2.0; IBM) software.

## 3 RESULTS

### 3.1 Patient and treatment characteristics

A total of 66 patients were eligible for inclusion in the study. Of these, 17 patients underwent PCI (PCI group) and 49 patients did not (non‐PCI group). At data cutoff (December 19, 2022), median follow‐up for OS was 16.05 months (inter quartile range [IQR] 10.08–20.89) for all patients (15.61 months [10.45–24.33] in the PCI group and 16.30 months [10.07–21.27] in the non‐PCI group). Among the two groups, the baseline characteristics of the patients were well balanced (Table 1). Overall, the median age was 64 years (IQR, 58–73), most patients were men (50 [75.76%] of 66), with an ECOG PS performance status of 0–1 (57 [86.36%]), and had a partial response to the initial treatment (60 [97%]). A total of 34 (51.52%) patients were smokers and 27 (40.91%) patients received consolidative thoracic radiotherapy.

**TABLE 1:** Baseline patient demographics and treatment characteristics.

Characteristic	PCI (*n* = 17)	non‐PCI (*n* = 49)	*t/χ^2^*	*p*‐value
Median age (range), years	68 (58–81)	62 (34–82)	1.70	0.09
Age group, years, *n* (%)			1.29	0.28
<65	7 (41.18)	28 (57.14)		
≥65	10 (58.82)	21 (42.86)		
Sex, *n* (%)			0.06	0.80
Male	12 (70.59)	38 (77.55)		
Female	5 (29.41)	11 (22.45)		
ECOG PS, *n* (%)			0.45	0.50
0–1	16 (94.12)	41 (83.67)		
2	1 (5.88)	8 (16.33)		
Smoker, *n* (%)			1.60	0.27
Yes	11 (64.71)	23 (46.94)		
No	6 (35.29)	26 (53.06)		
Response to initial treatment, *n* (%)			‐	0.64
Complete	2 (11.76)	4 (8.16)		
Partial	15 (88.24)	45 (91.84)		
Consolidative thoracic radiotherapy, *n* (%)			3.04	0.10
Yes	10 (58.82)	17 (34.69)		
No	7 (41.18)	32 (65.31)		

Abbreviations: PCI, prophylactic cranial irradiation; ECOG PS, Eastern Cooperative Oncology Group performance status.

The median number of atezolizumab or durvalumab cycles received was four (range, 3–28) in the PCI group and five (range, 4–21) in the non‐PCI group. The percentages of patients who received any subsequent systemic anticancer therapy in the two groups were 52.94% and 69.39%, respectively.

### 3.2 Overall survival analysis

**FIGURE 1:**
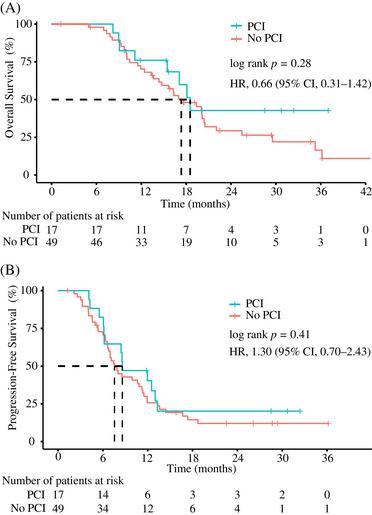
Kaplan‐Meier estimates of overall survival (A) and progression‐free survival (B) *Abbreviations*: PCI, prophylactic cranial irradiation; CI, confidence interval; HR, hazard ratio.

The Kaplan‐Meier survival curves for OS are shown in Figure 1A. OS was not significantly longer in the PCI group (median, 18.53 months; 95% CI, 16.23–20.83) than in the non‐PCI group (median, 17.35 months; 95% CI, 12.94–21.75). The stratified hazard ratio (HR) for death was 0.66 (95% CI, 0.31–1.42; *p* = 0.28). One‐year estimates of OS were 76.00% and 70.20% in the PCI and non‐PCI groups, respectively. In order to verify the association between the PCI status and OS, a multivariate Cox proportional hazards model was fitted with adjustments for other prognostic covariates, including sex (HR, 0.66; 95% CI, 0.30–1.49; *p* = 0.32), smoker (HR, 2.03; 95% CI, 1.00–4.12; *p* = 0.049), and consolidative thoracic radiotherapy (HR, 0.51; 95% CI, 0.26–1.03; *p* = 0.06). After multivariable adjustment, PCI was still not associated with significantly improved OS (HR, 0.68; 95% CI, 0.31–1.49; *p* = 0.34), as shown in Table 2.

**TABLE 2:** Univariate and multivariate Cox regression model for overall survival.

		Univariate analysis		Multivariate analysis	
Variables	*n*	HR (95% *CI*)	*p*‐value	HR (95% *CI*)	*p‐*valu*e*
Age (years)					
<65	35	Ref			
≥65	31	0.84 (0.45–1.54)	0.57		
Sex					
Male	50	Ref		Ref	
Female	16	0.50 (0.23–1.09)	0.08	0.66 (0.30–1.49)	0.32
ECOG PS					
0‐1	58	Ref			
2	8	1.60 (0.67–3.81)	0.29		
Smoker					
No	32	Ref.		Ref	
Yes	34	1.73 (0.92–3.23)	0.09	2.03 (1.00–4.12)	**0.049**
Response to initial treatment					
Partial	60	Ref			
Complete	6	0.49 (0.15–1.60)	0.24		
PCI					
No	49	Ref		Ref	
Yes	17	0.66 (0.31–1.42)	0.28	0.68 (0.31–1.49)	0.34
Consolidative thoracic radiotherapy					
No	39	Ref		Ref	
Yes	27	0.61 (0.33–1.14)	0.12	0.51 (0.26–1.03)	0.06

Abbreviations: CI, confidence interval; ECOG PS, Eastern Cooperative Oncology Group performance status; HR, hazard ratio; PCI, prophylactic cranial irradiation; Ref, reference.

### 3.3 Progression‐free survival analysis

At the data cutoff, 13 (76.47%) of the 17 patients in the PCI group and 41 (83.67%) of the 49 patients in the non‐PCI group had disease progression or died. Median PFS was also not significantly longer in the PCI group (8.61 months [95% *CI* 4.10–13.12]) than in the non‐PCI group (7.56 months [95% *CI* 6.15–9.00]; HR, 1.30 [95% *CI* 0.70–2.43]; *p* = 0.41; Figure 1B). At 6 and 12 months, the PFS rates in the PCI group were 76.50% and 40.30%, respectively, while those in the non‐PCI group were 70.70% and 25.70%, respectively. The univariate and multivariate models of PFS predictors are shown in Table 3. Univariate Cox regression analysis showed that none of the factors were significant prognostic indicators of PFS. Multivariate analysis showed that the factors associated with increased PFS were the absence of smoking and consolidative thoracic radiotherapy.

**TABLE 3:** Univariate and multivariate Cox regression model for progression‐free survival.

		Univariate analysis		Multivariate analysis	
Variables	*n*	HR (95% CI)	*p*‐value	HR (95% CI)	*p‐*valu*e*
Age (years)					
<65	35	Ref			
≥65	31	0.92 (0.54–1.57)	0.76		
Sex					
Male	50	Ref			
Female	16	0.68 (0.35–1.32)	0.25		
ECOG PS					
0‐1	58	Ref			
2	8	1.01 (0.43–2.35)	0.99		
Smoker					
No	32	Ref		Ref	
Yes	34	1.68 (0.98–2.89)	0.06	1.94 (1.11–3.38)	**0.02**
Response to initial treatment					
Partial	60	Ref			
Complete	6	0.69 (0.25–1.91)	0.47		
PCI					
No	49	Ref		Ref	
Yes	17	0.77 (0.41–1.44)	0.41	0.76 (0.40–1.44)	0.40
Consolidative thoracic radiotherapy					
No	39	Ref		Ref	
Yes	27	0.61 (0.35–1.07)	0.08	0.56 (0.31–1.00)	**0.048**

Abbreviations: CI, confidence interval; ECOG PS, Eastern Cooperative Oncology Group performance status; HR, hazard ratio; PCI, prophylactic cranial irradiation; Ref, reference.

### 3.4 Brain metastasis

**FIGURE 2:**
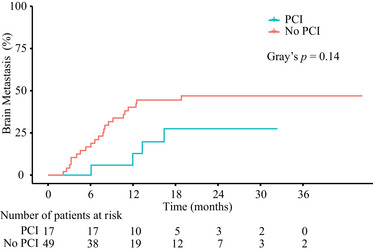
Cumulative incidence rate of brain metastases, treating death as a competing risk *Abbreviations*: PCI, prophylactic cranial irradiation.

Compared to the non‐PCI group, the cumulative incidence rate of BM was lower in the PCI group (12 and 18 months: 12.79% and 27.48% *vs*. 38.09% and 40.22%, respectively) when counting death as a competing risk, but the difference was not statistically significant (Gray's test, *p* = 0.14; Figure 2). Information about BM initial salvage treatment was as follows: salvage radiotherapy for BM was administered to one (25.00%) of four patients with BM in the PCI group and nine (45.00%) of 20 patients with BM in the non‐PCI group.

## 4 DISCUSSION

This first real‐world study aimed to analyze the feasibility of using PCI under active brain MRI surveillance for ES‐SCLC in the immunotherapy era and found that neither improvement in OS or PFS nor a decrease in the risk of developing BM was associated with PCI.

Notably, the median OS and PFS seen in this study were little longer than those observed in previous IMpower 133 (12.3 and 5.2 months) and CASPIAN phase III trials (13.0 and 5.1 months).^9^, ^10^ One possible reason could be that consolidative thoracic radiotherapy was not permitted in these phase III trials, whereas it was performed in 27 (40.91%) of 66 patients in our real‐world study. The results of the ASTRUM‐005 and CAPSTONE‐1 trials separately indicated that serplulimab and adebrelimab in combination with chemotherapy significantly improved the median OS (15.4 and 15.3 months, respectively) in the first‐line treatment of ES‐SCLC, with a good safety profile^13^, ^14^. Similarly, PCI was poorly controlled in the randomized trials mentioned previously. However, these two drugs were not included in our study because serplulimab and adebrelimab plus chemotherapy had not been approved for patients with ES‐SCLC prior to the initiation of this study.

In contrast to two randomized studies in Europe and Japan, PCI did not achieve a statistically significant difference in reducing BM in this study.^7^, ^8^ Immune checkpoint inhibitors that can penetrate the blood‐brain barrier may account for this observation. Atezolizumab prolonged the time to intracranial progression according to a subgroup analysis of the IMpower133 study.^15^ Moreover, durvalumab decreased the risk of central nervous system relapse in the Pacific Phase III NSCLC study.^16^ Therefore, PCI may be replaced by immune checkpoint inhibitors in the treatment of microscopic central nervous system diseases.^17^

Increased survival has led to greater concerns regarding long‐term nerve injury following PCI. Many studies have reported high frequencies of cognitive dysfunction and memory impairment after whole‐brain radiotherapy.^18^, ^19^, ^20^ MRI surveillance and salvage radiotherapy may have greater benefits for the cognitive function and quality of life than early radiation of the entire brain. Physicians should evaluate each patient's specific situation before providing comprehensive treatment to improve the survival and quality of life.

This study was limited by its retrospective nature, which means that selection bias and heterogeneity were inevitable among the enrolled patients. Second, given the small sample size and balanced baseline characteristics, propensity score matching was not performed to equalize the number of patients in the two groups and minimize selection bias. Third, as the toxicity of PCI and that associated with chest radiotherapy may be confounded, no toxicity‐related data were reported in this study.

## 5 CONCLUSIONS

The increasing use of brain MRI surveillance and immunotherapy for ES‐SCLC has further diminished the potential use of PCI in this population. Further studies are warranted to assess the efficacy and safety of PCI in patients with ES‐SCLC receiving immunotherapy.

## CONFLICT OF INTERESTS STATEMENT

There are no conflict of interests.

## ETHICS APPROVAL AND INFORMED CONSENT

All procedures involving human participants performed in this study were in accordance with the ethical standards of the local ethics committees (XYFY2022‐KL355‐01) and the 1964 Helsinki Declaration and its later amendments or comparable ethical standards.
